# Robotic parathyroidectomy is a feasible technique for primary hyperparathyroidism

**DOI:** 10.1007/s00423-023-03182-y

**Published:** 2023-12-16

**Authors:** Jin Seok Lee, Jun Sung Lee, Hojung Jeong, Hyeok Jun Yun, Hojin Chang, Seok Mo Kim, Yong Sang Lee, Hang-Seok Chang

**Affiliations:** Department of Surgery, Thyroid Cancer Center, Institute of Refractory Thyroid Cancer, Gangnam Severance Hospital, Yonsei University College of Medicine, 211 Eonjuro, Gangnam-Gu, Seoul, 135-720 Korea

**Keywords:** Primary hyperparathyroidism, Parathyroidectomy, Robot surgery

## Abstract

**Purpose:**

Focused parathyroidectomy is the gold standard treatment modality for primary hyperparathyroidism, which allows accurate preoperative localization. Robotic parathyroidectomy has emerged as a feasible procedure for focused parathyroidectomy. This study aimed to report the experiences of gasless robotic transaxillary parathyroidectomy for primary hyperparathyroidism in a single center.

**Methods:**

We assessed the data obtained from patients who underwent gasless robotic parathyroidectomy with the transaxillary approach between December 2013 and August 2022 and were diagnosed with primary hyperparathyroidism at our institute. The data included clinical, biochemical, and pathological features and operation time.

**Results:**

Of the 12 patients, 11 were women and one was a man. The median age of the patients was 44.5 years (range: 15–65 years). The median preoperative maximum mass diameters on ultrasonography and neck computed tomography were 1.2 ± 0.5 and 1.1 ± 0.6 cm, respectively. The median size of the postoperative maximum mass diameter in gross pathology was 1.3 ± 0.4 cm. The location of the enlarged parathyroid was left superior in five patients, right inferior in four, left inferior in three, and no right superior in one. In the final pathological examination, all cases were parathyroid adenomas. Only one case experienced a postoperative bleeding complication. At six months from surgery, average of an axillary scar length was 5.85 cm, and an average width was 0.21 cm. The mean operative time was 113 ± 48 min. The mean robot docking and console times were 9 ± 5 and 47 ± 52 min, respectively.

**Conclusions:**

Robotic transaxillary parathyroidectomy is a feasible technique in select patients with primary hyperparathyroidism and preoperatively localized disease. The gasless robotic transaxillary approach provides procedural safety as well as superior cosmetic results without a neck scar.

## Introduction

Focused parathyroidectomy is the most effective therapy for localized primary hyperparathyroidism (PHPT). The history of parathyroidectomy dates back to 1925, when Felix Mandel performed the first successful parathyroid surgery  [1]. Until the late 1970s, the standard approach to parathyroidectomy was a four-gland exploration that was performed under general anesthesia using a large skin incision  [2]. In the early 1980s, some studies began evaluating the success of parathyroidectomy without evaluating all four parathyroid glands. More than 80% of patients with PHPT have a single-gland disease. Unilateral neck exploration and elective or focused parathyroidectomy (involving one gland) have been proposed since the introduction of improved preoperative localization examination methods such as ultrasonography, sestamibi scanning, and intraoperative parathyroid hormone (IOPTH) monitoring  [3, 4]. The cure and complication rates are equivalent to those of the conventional open approach. The advantages include reduced operating time, smaller scars, shorter hospitalization durations, and less postoperative analgesic requirement  [5]. Focused exploration is currently the standard method for parathyroidectomy worldwide  [2, 4, 6, 7].

Various approaches to avoid neck scarring through minimally invasive or remote incisional access have been developed for parathyroidectomy  [8]. In particular, the gasless robotic transaxillary approach to the thyroid gland and subsequently to the parathyroid glands was developed in South Korea. Surgeons in the USA and Europe who have adopted robotic surgery have published various case reports and studies  [8, 9]. This study aimed to report the successful experiences of a single surgeon who performed gasless robotic transaxillary parathyroidectomies for PHPT in a single center, safely.

## Methods

**Fig. 1:**
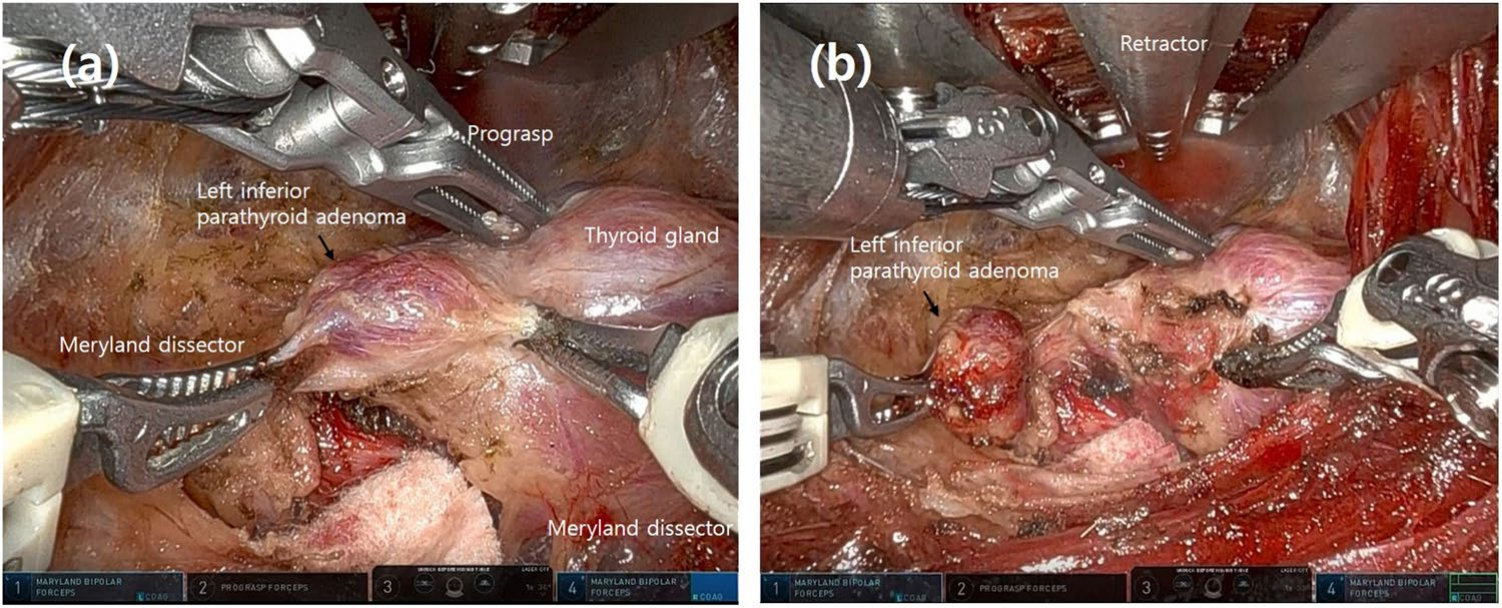
Robotic transaxillary parathyroidectomy for a patient with focused primary hyperparathyroidism. **a** An intraoperative view after dissecting left inferior parathyroid adenoma. **b** After removing the left inferior parathyroid gland

Twelve patients diagnosed with PHPT underwent gasless robotic parathyroidectomy with the transaxillary approach at the Gangnam Severance Hospital between December 2013 and August 2022. The indications for robot transaxillary parathyroidectomy were not significantly different from those for conventional parathyroidectomy. The target group includes individuals with symptomatic primary hyperparathyroidism suspected to have parathyroid adenoma or hyperplasia, who have not experienced any specific neck injuries or previous surgeries, and who have consented to bear the higher surgical costs. Medical records of these 12 patients were reviewed retrospectively, including data on demographic, clinical, imaging, and laboratory characteristics and intraoperative techniques with operation time. The laboratory data collected included levels of parathyroid hormone (PTH) and serum calcium with reference ranges of 15–65 pg/mL and 8.5–10.1 mg/dL, respectively. Pathological data were reviewed by a pathologist using a specimen slide. Imaging data were collected, including ultrasound (US), neck computed tomography (CT), and sestamibi scans, for preoperative localization. The operation targeted patients who wanted to undergo robotic surgery. Patients who had previous neck surgery, neck irradiation, neck mobility problems, cervical spine problems, and suspected parathyroid carcinoma were excluded. The surgical technique used was based on robotic transaxillary thyroidectomy. Except for skin marking with preoperative neck US, the surgical technique was similar to thyroidectomy from skin incision to docking of the robot arms. The thyroid glands were first identified and dissected. A parathyroid adenoma was then identified and resected (Fig. 1). Operative time was defined as the time from incision to skin closure. Docking time was defined as the time from the insertion of the retractor to the start of console time. Console time was defined as the time of operation using a robot. The success of the treatment was determined biochemically by normalization of the serum calcium and PTH levels at a minimum of 2 weeks following surgery and by histopathological confirmation of the presence of hypercellular parathyroid tissue in the excised lesion.

## Results

**Fig. 2:**
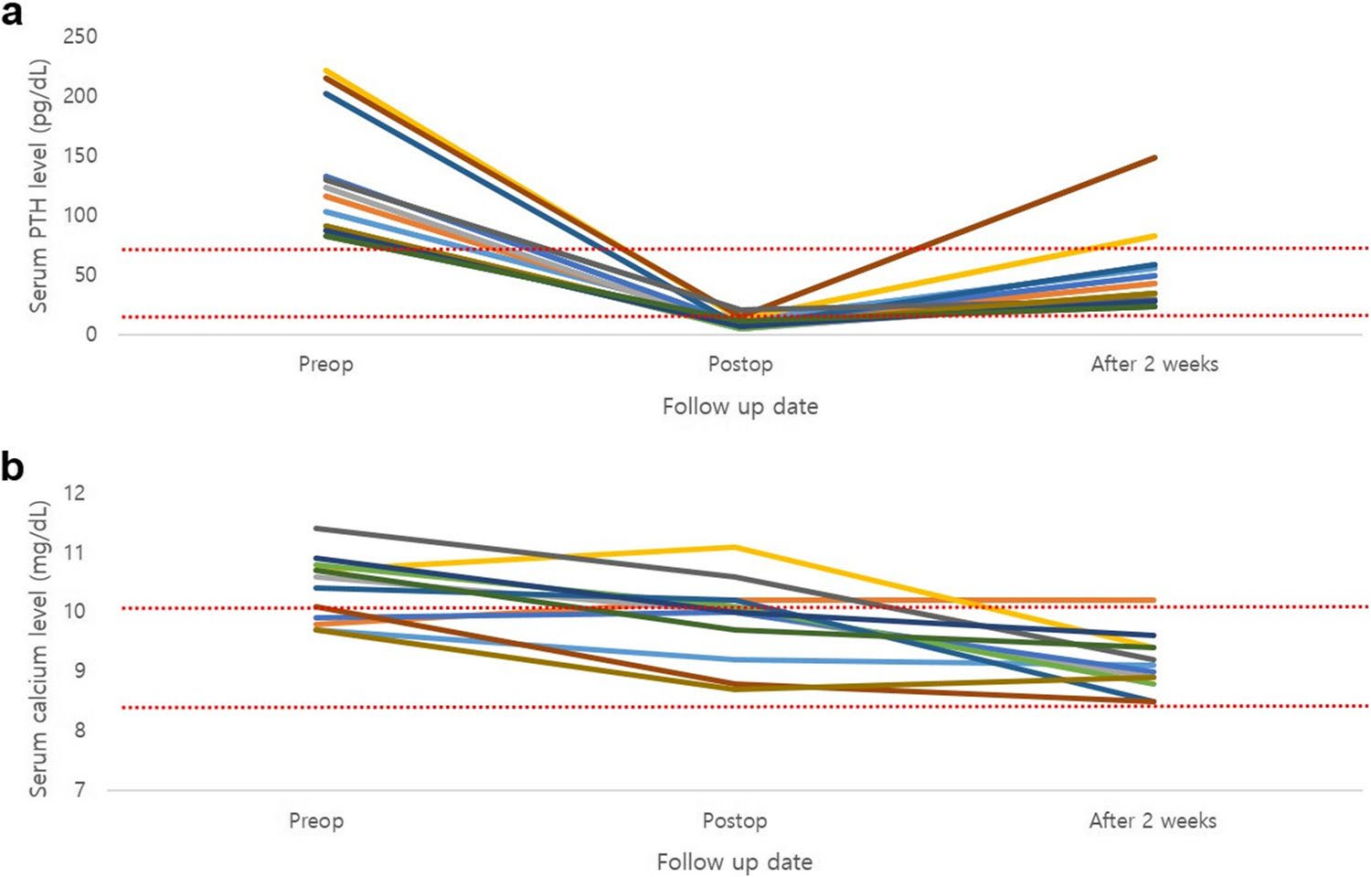
Pre- and postoperative serum levels of (**a**) parathyroid hormone (PTH) and (**b**) calcium of patients with primary hyperparathyroidism. Between red-dotted lines are normal range

Of the 12 patients, 11 were women and one was a man. The median age of the patients was 44.5 years (range:15–65 years). Demographic and clinicopathological features are explained in Table 1. All patients showed high preoperative PTH levels that were higher than their normal range (range:83.2–222.4 pg/mL). Serum PTH levels were measured 8 h after surgery and showed a decrease by more than 20% compared to the preoperative serum PTH levels. Initial normalization of PTH levels occurred in 10 patients and was seen 2 weeks after surgery. Among the two cases that exhibited postoperative high PTH levels, one showed higher serum PTH levels measured 15 months after surgery, with a decreasing tendency (149.1 > 115.0 > 112.0 > 101.0 pg/mL). Eight cases showed high serum calcium levels before surgery. Two weeks after surgery, normalization of serum calcium levels occurred in 11 cases, and one had a slightly high serum calcium level (10.2 mg/dL) with normal serum PTH levels. Serum PTH and calcium levels were monitored for 2 weeks across internals in outpatient clinics (Fig. 2). For preoperative localization, ultrasonography, neck CT, and sestamibi scans were performed. The location of the focused parathyroid was five left superior, four right inferior, three left inferior, and none right superior (Table 1). The median size of maximum preoperative mass diameter in US and neck CT was 1.2 ± 0.5 and 1.1 ± 0.6 cm, respectively. The median postoperative maximum mass diameter in the final pathology was 1.3 ± 0.4 cm. In the final pathological examination, all cases were confirmed as parathyroid adenomas.

**Table 1:** Demographics and clinicopathological features of 12 primary hyperparathyroidism patients

Case ID	Sex (F: female, M: male)	Age (years)	Serum PTH level (15–65 pg/mL)			Serum calcium level (8.5–10.1 mg/dL)			Preoperative localization	Pathology	Maximum diameter (cm)		
			Preop	Postop	After 2 weeks	Preop	Postop	After 2 weeks			US	CT	Pathologic report
1	F	39	103.2	12.6	55.8	9.7	9.2	9.1	Left superior	Adenoma	1.2	1.0	1.2
2	F	48	116.4	7.6	42.9	9.8	10.2	10.2	Right inferior	Adenoma	1.8	2.1	1.4
3	F	49	123.7	5.1	29.5	10.6	10.0	8.9	Left inferior	Adenoma	-	0.6	1.0
4	F	47	222.4	13.2	82.8	10.7	11.1	9.4	Right inferior	Adenoma	1.2	1.2	1.1
5	F	49	133.3	8.5	49.1	9.9	10.0	9.0	Left superior	Adenoma	1.9	1.0	1.8
6	F	42	91.3	4.7	34.3	10.8	10.1	8.8	Left superior	Adenoma	0.9	0.8	0.9
7	F	41	202.3	7.0	59.0	10.4	10.2	8.5	Left inferior	Adenoma	1.8	-	1.0
8	F	38	215.5	15.4	149.1	10.1	8.8	8.5	Left superior	Adenoma	2.3	2.4	2.2
9	M	39	130	20.7	28.9	11.4	10.6	9.2	Right inferior	Adenoma	0.8	0.8	1.5
10	F	51	91.5	7.6	34.4	9.7	8.7	8.9	Right inferior	Adenoma	1.7	1.6	1.7
11	F	65	87.5	7.1	27.9	10.9	10.0	9.6	Left superior	Adenoma	1.6	1.2	1.7
12	F	15	83.2	11.0	23.7	10.7	9.7	9.4	Left inferior	Adenoma	1.1	-	1.0

PTH, pg/mL; serum calcium, mg/dL

*PTH* parathyroid hormone, *US* ultrasonography, *CT* computed tomography

Out of 12 cases, only one case experienced a post-operative bleeding complication (Table 2). The patient was suspected to have flap bleeding on the pectoralis major area on POD#1. After compression with elastic bandage on the swollen area, no further bleeding occurred, and the patient was observed without invasive procedures like hematoma evacuation. Furthermore, there were no complications such as jugular vein injury that can occur during transaxillary robot thyroidectomy/parathyroidectomy. At six months from surgery, axillary scar length and width were measured, with an average length of 5.85 cm and an average width of 0.21 cm (Table 2).

**Table 2:** Complications and axillary scar of transaxillary robotic parathyroidectomies

	Total cases (*n* = 12)
Complications, *n* (%)	1 (8.3)
RLN palsy	0 (0)
Bleeding	1 (8.3)
Jugular vein injury	0 (0)
Wound infection	0 (0)
Hypocalcemia	0 (0)
Scar, mean ± SD, cm	
Length	5.85 ± 0.50
Width	0.21 ± 0.06

*RLN* recurrent laryngeal nerve, *SD* standard deviation

In the intraoperative situation, the mean operation time was 113 ± 48 min (Table 3). The mean robot docking and console times were 9 ± 5 and 47 ± 52 min, respectively. One patient (Case 7) with an operative time of over 200 min had multiple enlarged parathyroid adenoma-like masses in the left lower quadrant; one of which was found to be metastatic papillary thyroid carcinoma on frozen pathology. A robotic transaxillary thyroid lobectomy with focused parathyroidectomy was performed, and a parathyroid adenoma with intrathyroidal papillary thyroid microcarcinoma was identified in the final pathologic reports. The average estimated blood loss during robot transaxillary parathyroidectomy was approximately 30 mL. This was achievable because there were no significant vessel injuries during surgery, and it was possible due to the well-established procedures.

**Table 3:** Operation, docking, and console time of transaxillary robotic parathyroidectomies

Case ID	Operation time	Docking time	Console time
1	105	10	5
2	61	4	10
3	101	3	57
4	119	20	50
5	81	12	28
6	142	5	67
7	223	6	173
8	78	10	10
9	108	10	25
10	177	6	36
11	116	6	14
12	90	6	24

## Discussion

A parathyroid adenoma can be removed without visualizing the other glands if it has been well-localized preoperatively using imaging studies. The focused method achieved the same results as those obtained after bilateral neck exploration with four-gland visualization. Therefore, minimal approach techniques have replaced bilateral neck exploration in patients with localized PHPT, although open neck exploration remains the best surgical approach for nonlocalized PHPT  [10].

The robotic transaxillary approach has many advantages for many types of thyroid surgery that have been previously reported in the literature. It allows three-dimensional visualization that provides more perceptual depth and magnification, allowing for precise tissue dissection. Within a limited working space, such as the anterior neck or superior mediastinum, it permits precise parathyroid surgery with free-moving robot arms in a small space  [11–14]. As documented by Ismail et al.  [15], this approach to the superior mediastinum allowed robotic removal of ectopic thyroid glands. No requirement for carbon dioxide insufflation is one of the advantages of this approach  [12, 16]. Furthermore, the main advantage of the transaxillary approach is that no visible scars are formed on the neck. Scars on easily visible parts of the body such as the anterior neck have a detrimental effect on body image and are perceived as worse than scars that can be hidden by clothing  [17, 18]. Eye-tracking technology has shown that scars on the neck are noticeable enough to draw visual attention from the face to the scar area  [19, 20].

The limitations of robot-assisted approaches for neck surgery documented in the literature include the need for strict patient selection criteria, experienced surgeons, longer operation time than those of open surgeries, and higher surgical costs  [12, 16]. One of the unique limitations of the robotic transaxillary approaches is the complications associated with the flap because a larger subcutaneous tissue dissection area is required with this approach. Studies have shown contradictory results in the case of surgical site pain with a large flap. Some reported lesser pain with the robot-assisted approach, and others reported mild postoperative symptoms associated with the flap, including pain and paresthesia  [12].

The critical limitation of this approach is the inability to perform a bilateral exploration because of inadequate contralateral exposure  [11]. Therefore, this limitation should be discussed with the patients, and it should be emphasized that in case the intraoperative PTH levels do not decrease as expected, they would need conventional open neck exploration surgery.

Short-term complications, such as recurrent laryngeal nerve palsy, bleeding, hypocalcemia, or wound infection, were not observed in this study. In two patients, the postoperative PTH levels measured 2 weeks after surgery were elevated with normal serum calcium levels. One of these patients (case 8) exhibited elevated serum PTH levels even after 15 months of surgery, though the levels of PTH showed decreasing tendency (149.1 > 115.0 > 112.0 > 101.0 pg/mL). No suspicious recurrence or contralateral parathyroid adenoma was observed in the US imaging study 15 months after surgery. Because the patient showed no symptoms and serum calcium levels were normal, a follow-up after 3 months was planned.

The mean operation time in this study was 113 ± 48 min, which is shorter than that reported previously  [6, 11]. The operation time showed a progressive reduction in total operation time from over 5 h to under 1 h in other studies  [13, 21]. Various factors can affect the total operative time, including familiarity of the entire operating room team with the procedure because the teams which perform a large number of these cases will become familiar with the equipment and processes involved  [21]. In this series, one surgeon who had performed more than 500 robotic transaxillary thyroidectomies performed robotic parathyroidectomy; therefore, he did not have a steep learning curve. One patient (case 7) had multiple enlarged masses resembling a parathyroid adenoma in the left inferior area, one of which revealed metastatic papillary thyroid carcinoma on frozen pathology. A robotic transaxillary thyroid lobectomy with focused parathyroidectomy was performed, and a parathyroid adenoma with intrathyroidal papillary thyroid microcarcinoma was identified in the final pathologic reports.

In conclusion, robotic transaxillary parathyroidectomy is a feasible technique in select patients with PHPT and preoperatively localized disease. The gasless robotic transaxillary approach provides procedural safety as well as superior cosmetic results without a neck scar.

## Data Availability

The data that support the findings of this study are available from the corresponding author, YL, upon reasonable request.
